# Reviewer acknowledgement 2013

**DOI:** 10.1186/1472-6920-14-24

**Published:** 2014-02-10

**Authors:** Fernando Marques

**Affiliations:** 1 BioMed Central, 236 Gray's Inn Road, WC1X 8HB, London

## Abstract

**Contributing reviewers:**

The Editors of *BMC Medical Education* would like to thank all our reviewers who have contributed to the journal in Volume 13 (2013).

## 

Olaf Aasland

Norway

Francis Achike

USA

Srikar Adhikari

USA

Dimitri Aerden

Belgium

Sourabh Aggarwal

India

Rola Ajjawi

UK

Jill Allison

Canada

Kenton Anderson

USA

Kathryn Andolsek

USA

Allen Andrade

USA

Jane Andreasen

Denmark

Anne Arber

UK

Cassandra Arceneaux

USA

Julian Archer

UK

Natasha Archer

USA

Juan Antonio Armenta Peinado

Spain

Louise Arnold

USA

Rahul Arya

USA

Sara Ashencaen Crabtree

UK

Tobey Audcent

Canada

Samy Azer

Saudi Arabia

Kaukab Azim

Saudi Arabia

Jonathan Bae

USA

Samira Bamuhair

Saudi Arabia

Rebecca Barnes

UK

Hugh Barr

UK

David Bearn

UK

David Bender

UK

Tanya Beran

Canada

Alicia Bergman

USA

Aaron Bernard

USA

Viviane Bernardo

Brazil

Henning Biermann

Germany

Saad M. Bindawas

Saudi Arabia

Natalie Blencowe

UK

Marcus Bloice

Austria

Amy Blue

USA

Phil Blyth

New Zealand

Valdes Bollela

Brazil

Thomas Booth

UK

Miles Bore

Australia

Jan Borleffs

Netherlands

Gisele Bourgeois-Law

Canada

Judith Bowen

USA

Andrew Bowhay

UK

Matt Boyd

New Zealand

Isobel Braidman

UK

Birgitte Brandstrup

Denmark

Richard Branstrom

Sweden

Jarle Breivik

Norway

Emily Brennan

USA

Andrew Brittlebank

UK

Doug Brock

USA

William Brooks

USA

Jo Brown

UK

Ryan Brydges

Canada

David Buck

USA

Sharon Buckley

UK

Bryan Burford

UK

Julius Burkauskas

Lithuania

Aidan Byrne

UK

Jeff Cain

US Minor Outlying Islands

Gordon Caldwell

UK

Brian Cameron

Canada

Craig Canby

USA

Peter Cantillon

Ireland

Rosemary Caron

USA

Sandra Carr

Australia

Donald Casey

USA

Afonso Cavaco

Portugal

Judith Cave

UK

Penny Cavenagh

UK

Juan Cendan

USA

Samantha Chakraborty

Austria

Pratap Challa

USA

Madawa Chandratilake

Sri Lanka

Xu Chaojin

China

Bernard Charlin

Canada

Andrew Chaytor

UK

Belinda Chen

USA

Lucy Chipchase

Australia

Anna Chisholm

UK

Maria Chun

USA

Phillip Clark

USA

Jennifer Cleland

UK

Hamish Coates

Australia

Deborah Cohen

UK

Janke Cohen-Schotanus

Netherlands

Fabrizio Consorti

Italy

Nick Cooper

UK

Katherine S. Corker

USA

Patrício Costa

Portugal

Philip Cotton

UK

Paul Crampton

UK

Trevor Crowe

Australia

Monica Cuddy

USA

Shauna Cunningham

UK

Fiona Curtis

UK

Anne Cushing

UK

Eugene Custers

Netherlands

Jane Dacre

UK

Prithwiraj Das

UK

David Davies

UK

Dave Davis

USA

Joan Mary Davis

USA

Penelope Davis

Canada

Angela Dawson

Australia

Tjalling de Vries

Netherlands

Bram De Wever

Belgium

Benedicte De Winter

Belgium

Matthew DeCamp

USA

Clare Delany

Australia

Allard Dembe

USA

Olufemi Desalu

Nigeria

Vasudha Devi

India

Marie-Louise Dick

Australia

Peter Dieter

Germany

Agnes Dodds

Australia

Diana Dolmans

Netherlands

Benjamin Doolittle

USA

Tim Dornan

Netherlands

David Doukas

USA

Athanassios Douzenis

Greece

Susan Dovey

New Zealand

James Dunbar

Australia

Roger Dunston

Australia

Herman Düsman

Netherlands

Oliver James Dyar

UK

Gudrun Edgren

Sweden

Luzie Eisleben

Germany

Abdel-Hady El-Gilany

Egypt

Edzard Ernst

UK

Nermin Ersoy

Turkey

Christopher Etherton-Beer

Australia

Catrin Evans

UK

Glen Farr

USA

Ramon Fayad

Colombia

Christopher Feddock

USA

Gabrielle Finn

UK

Edward Finnerty

USA

Eoin Flanagan

USA

Lisa Fleet

Canada

Uno Fors

Sweden

John Frain

UK

Maureen Francis

USA

Jose Francois

Canada

Erica Frank

Canada

Kristin Fraser

Canada

Adrian Freeman

UK

Alexander Fuld

USA

David Gachoud

Switzerland

Jan Gaertner

Germany

Robert Gagnon

Canada

Tom Gale

UK

Jenny Gallagher

UK

Pallab Ganguly

Saudi Arabia

Paul Garrud

UK

Paul Gavaza

USA

Raewyn Gavin

New Zealand

Eileen Giles

Australia

Ronald Gimbel

USA

Helenice Gobbi

Brazil

Michael Goldacre

UK

Helen Goodyear

UK

Andrew Grant

UK

Barbara Griffin

Australia

Charles Griffith

USA

Reinou Groen

USA

Frans Grosfeld

Netherlands

Michele Groves

Australia

Larry Gruppen

USA

Anthony Guerrero

USA

Dilek Guldal

Turkey

Hongfei Guo

USA

Erol Gurpinar

Turkey

Antonio Gutierrez

USA

Jose Gutierrez-Maldonado

Spain

Fawzia Habib

Saudi Arabia

Julie Hadley

UK

Harry Haigler

USA

Stuart Haines

USA

Brigette Hales

Canada

Andrew Hall

UK

Hossam Hamdy

United Arab Emirates

Wolfgang Hampe

Germany

Karin Hannes

Belgium

Mark Harbinson

UK

Sigrid Harendza

Germany

Neil Harrison

UK

Don Harting

USA

Iman Hegazi

Australia

Phil J.M. Heiligers

Netherlands

Gordon Hendry

Australia

Marcus Henning

New Zealand

Carol Herbert

Canada

Amir Herman

Israel

Karen Heskett

USA

Mariana Hewson

USA

Andrew Hill

New Zealand

Achim Hochlehnert

Germany

Are Holen

Norway

Sean Hood

Australia

Wendy Hu

Australia

Julia Huber

Germany

Judith Hudson

Australia

Neil Hudson

UK

Gerald Humphris

UK

Mark Huntington

USA

Sören Huwendiek

Switzerland

Dragan Ilic

Australia

Jan Illing

UK

Marita Rohr Inglehart

USA

Laili Irani

USA

Pekka Isotalus

Finland

Debra Jackson

Australia

Hanna Järvenoja

Finland

Sudha Jayaraman

USA

Saroj Jayasinghe

Sri Lanka

Peter Jaye

UK

Suh-Fang Jeng

Taiwan

Morten Lind Jensen

Denmark

Farahnaz Joukar

Iran

Tina Dewi Judistiani

Indonesia

Jana Juenger

Germany

Jonas Jurgaitis

Lithuania

Alicja Juskiene

Lithuania

Natan Kahan

Israel

John Kalbfleisch

USA

Elsbeth Kalenderian

USA

Gurvinder Kalra

India

Robert Kamei

Singapore

Carol Kamin

USA

Filiz Kantek

Turkey

Hans Karle

Denmark

Orit Karnieli-Miller

Israel

Aliya Kassam

Canada

Hitomi Kataoka

Japan

Milica Katic

Croatia

Argyro Kavadella

Greece

Nurten Kaya

Turkey

Clare Kell

UK

Patricia J Kelly

USA

Jean Ker

UK

Shweta Khandelwal

India

Sharla King

Canada

Karl Kingsley

USA

Matthias Knobe

Germany

Jennifer Kogan

USA

Joseph Kolars

USA

Lars Konge

Denmark

Chris Krägeloh

New Zealand

Anneke Kramer

Netherlands

Sunčana Kukolja

Croatia

Jan Kuks

Netherlands

Arno Kumagai

USA

Suzanne Kurtz

USA

Viji Kurup

USA

Rashmi Kusurkar

Netherlands

Timothy Lahey

USA

Nai Ming Lai

Malaysia

Anita Laidlaw

UK

Fiona Lake

Australia

Trevor Lambert

UK

Constantin Landes

Germany

Xiang Qian Lao

Hong Kong

Judith Lathlean

UK

Matthew Leach

Australia

Young-Mee Lee

Korea South

Jeremie Lefevre

France

Ronny Lehmann

Germany

Sam Leinster

UK

Jeff Leiter

Canada

Heidi Lempp

UK

Sum Leong

Ireland

Laurent Letrilliart

France

Elise Lev

USA

Leah Levi

USA

Linda Lewin

USA

Frank Lewis

UK

Jie Liang

China

Robert Likic

Croatia

Chaou-Shune Lin

Taiwan

Stefan Lindgren

Sweden

Gill Livingston

UK

Andrew Lockey

UK

Yoon Kong Loke

UK

Naty Lopez

USA

Suzana Loshkovska

Macedonia

Gaetano Lotrecchiano

USA

Antonella Lotti

Italy

David Lowe

UK

Sofie Loyens

Netherlands

Stuart Lubarsky

Canada

Susanna Lucieer

Netherlands

Jo Lunn Brownlee

Australia

Andrew Luxton-Reilly

New Zealand

Joanne Lymn

UK

Irene Ma

Canada

Martin MacDowell

USA

Ajay Mahal

Australia

Rathi Mahendran

Singapore

Meredith Makeham

Australia

Sadia Malick

UK

Bente Malling

Denmark

Judy Mannix

Australia

Barrie Margetts

UK

Tina Martimianakis

Canada

Matko Marusic

Croatia

Ken Masters

Oman

Win May

USA

Annie McCluskey

Australia

Geoffrey McColl

Australia

Christopher McCoy

USA

Elizabeth McGann

USA

Matthew McGrail

Australia

John McGreevey

USA

Karen McKelvie

UK

Danette McKinley

USA

Chris McManus

UK

John McNulty

USA

Alan Merry

New Zealand

Sylvia Metcalfe

Australia

Felise Milan

USA

Steve Milanese

Australia

Susan Miles

UK

David Miller

USA

Clinton Mitchell

New Zealand

Spiros Miyakis

Australia

Charles Mkony

Tanzania

Susan Moch

USA

Juraj Mokry

Slovakia

Willemina Molenaar

Netherlands

Antonio Moreira

Portugal

Ann Morrison

USA

Torbjørn Åge Moum

Norway

Don Munro

Australia

Deborah Murdoch-Eaton

UK

Thirusha Naidu

South Africa

Susan Nancarrow

Australia

Corina Naughton

UK

Brett Nelson

USA

Bruce Newton

USA

Sandra Nicholson

UK

Christoph Nikendei

Germany

Janneke Noordman

Netherlands

Jonas Nordquist

Sweden

Cristiana Nunes

Brazil

Aileen O'Brien

UK

Elke Birgit Ochsmann

Germany

Nina Rydland Olsen

Norway

Carole Orchard

Canada

Jay Orlander

USA

Christoph Ostgathe

Germany

Nilgun Ozcakar

Turkey

Alvisa Palese

Italy

Edward Palmer

Australia

Xiao-chuan Pan

China

Maria Papadakaki

Greece

Lazaros Papadopoulos

Greece

Alexia Papageorgiou

UK

Andrew Papanikitas

UK

Tracey Papinczak

Australia

Rona Patey

UK

Adam Paul

UK

Wojciech Pawlina

USA

Alan Pearson

Australia

Burcin Pehlivanoglu

Turkey

Godfrey Pell

UK

Adriana Perez

USA

David Perkins

Australia

Susan Persky

USA

Ray Peterson

Australia

Christine Phillips

Australia

Victoria Phillips

USA

Jan L Plass

USA

Kathryn Pollak

USA

Phillippa Poole

New Zealand

Jason Post

USA

Tamara Power

Australia

Gill Price

UK

Sue Pullon

New Zealand

Jennifer Quaintance

USA

Natalie Radomski

Australia

Daryl Ramai

Grenada

Fernando Ramos

Portugal

Patangi Rangachari

Canada

Gopinath Ranjith

UK

Nyapati Rao

USA

Linda Raphael

USA

Tobias Raupach

Germany

Gilbert Reibnegger

Austria

Susan Rhind

UK

Christopher Ricketts

UK

Josef Rieder

Austria

Charlotte Ringsted

Denmark

Jonathan Ripp

USA

Christopher Roberts

Australia

Martin Roberts

UK

William Roberts

USA

Doug Rohrer

USA

Marcy Rosenbaum

USA

Jerome Rotgans

Singapore

Charlotte Rothwell

UK

Christine Rubie-Davies

New Zealand

Gudrun Rudolfsson

Sweden

Kenneth Ruit

USA

Gerard Rushton

USA

Sivalal Sadasivan

Malaysia

Serafin Sanchez Gomez

Spain

John Sandars

UK

Kathryn Sanders

USA

Sally Santen

USA

Emmanuel Sanya

Nigeria

Antonio Sarikas

Germany

Marina Sawdon

UK

Lydia Schaap

Netherlands

Stefan K. Schauber

Germany

Albert Scherpbier

Netherlands

Ralf Schmidmaier

Germany

Julie Scholes

UK

Michael Schreiber

Germany

Sebastian Schubert

Germany

Carl Schulman

USA

Georgina Selby

UK

Nick Sevdalis

UK

Patricia Sexton

USA

Naiemeh Seyedfatemi

Iran

Nachiket Shankar

India

Ravi Pathiyil Shankar

Nepal

Judy Shea

USA

Amir Shmueli

Israel

Tim Shortus

Australia

Charles Shuler

Canada

Victor Sierpina

USA

Jonathan Silverman

UK

Anne Simmenroth-Nayda

Germany

Rich Simpson

USA

Susan Skledar

USA

David Sleet

USA

Claire Smith

UK

Megan Smith

Australia

Pamela Smithburger

USA

Alisdair Smithies

UK

Suzanne Snodgrass

Australia

Jean-Karl Soler

Malta

Rita Sood

India

Gina Sorrentino

USA

John Spangler

USA

Jeffrey Spike

USA

Cord Spreckelsen

Germany

Sue Steele

UK

Tomasz Stefaniak

Poland

Karen Stegers-Jager

Netherlands

Terese Stenfors-Hayes

Sweden

Fred Stevens

Netherlands

Michael Stevenson

UK

Kevin Stirling

UK

Christiane Stock

Denmark

Hugh Stoddard

USA

Emma Stodel

Canada

Peter Stone

New Zealand

Ghadeer Suaifan

Jordan

Winnie Suen

USA

Eriko Sumi

Japan

Johanne Sundby

Norway

Tarja Suominen

Finland

Igor Svab

Slovenia

Angela Taft

Australia

Masami Tagawa

Japan

Ann Taket

Australia

Marie Tarrant

Hong Kong

Kimberly Tartaglia

USA

David Taylor

UK

Myra Taylor

South Africa

Dirk Tempelaar

Netherlands

Olle Ten Cate

Netherlands

Daniel Terry

Australia

Pim Teunissen

Netherlands

Andrea Thompson

New Zealand

Tobias Todsen

Denmark

Martin Tolsgaard

Denmark

Malvin Torsvik

Norway

Andrea Trevisan

Italy

John Triano

Canada

Marc Triola

USA

Philip Tucker

UK

Vicki Tully

UK

Sevgi Turan

Turkey

Oktay Tutarel

Germany

Mike Tweed

New Zealand

Lynda Tyer-Viola

USA

Reidar Tyssen

Norway

Christine Urquhart

UK

Alfonso Urzúa

Chile

Paula Vainiomäki

Finland

Philip van der Wees

Netherlands

Philip van Eijndhoven

Netherlands

Thed van Leeuwen

Netherlands

Paul Van Royen

Belgium

Marta van Zanten

USA

Mark Vander Weg

USA

Shawn Varney

USA

Nagaswami Vasan

USA

Anna Vnuk

Australia

Karen Voigt

Germany

Deepti Vyas

USA

Maureen Waine

Australia

Allyn Walsh

Canada

Stewart Walsh

Ireland

Ulla Walter

Germany

James Ware

Saudi Arabia

Alison Warren

Ireland

Simon Watmough

UK

Andy Wearn

New Zealand

Lynn Weekes

Australia

Jennifer Mary Weller

New Zealand

Xiaozhong Wen

USA

Gig Wernbacher-Searle

Austria

Anne Werner

UK

Colin West

USA

Kath Weston

Australia

Maria Weurlander

Sweden

Peter Weyrich

Germany

Susan Whicker

Australia

Charles Wiles

UK

Matthew Wiles

UK

Ann Wilkinson

UK

Tim Wilkinson

New Zealand

Marie Williams

Australia

Ian Wilson

Australia

Donna Windish

USA

Christopher Wittich

USA

Ruth Wong

UK

Stanley Wong

USA

Michael Woo

Canada

Katherine Woolf

UK

Glenda Wrenn

USA

Scott Wright

USA

Tsu Yin Wu

USA

Naohito Yamaguchi

Japan

Janet Yates

UK

Michael Yelland

Australia

Louise Young

Australia

George Yousef

Canada

Farid Youssef

Trinidad and Tobago

Tzu-Chieh Yu

New Zealand

Muhamad Saiful Bahri Yusoff

Malaysia

Xiulan Zhang

China

Yuhai Zhang

China

Sanjay Zodpey

India

## Pre-publication history

The pre-publication history for this paper can be accessed here:

http://www.biomedcentral.com/1472-6920/14/24/prepub

